# A Machine Learning Based Framework to Identify and Classify Non-alcoholic Fatty Liver Disease in a Large-Scale Population

**DOI:** 10.3389/fpubh.2022.846118

**Published:** 2022-04-04

**Authors:** Weidong Ji, Mingyue Xue, Yushan Zhang, Hua Yao, Yushan Wang

**Affiliations:** ^1^Department of Medical Information, Zhongshan School of Medicine, Sun Yat-sen University, Guangzhou, China; ^2^Hospital of Traditional Chinese Medicine Affiliated to the Fourth Clinical Medical College of Xinjiang Medical University, Urumqi, China; ^3^Department of Maternal and Child Health, School of Public Health, Sun Yat-sen University, Guangzhou, China; ^4^Center of Health Management, The First Affiliated Hospital of Xinjiang Medical University, Urumqi, China

**Keywords:** machine learning, screening model, LASSO, non-alcoholic fatty liver disease (NAFLD), predictive models

## Abstract

Non-alcoholic fatty liver disease (NAFLD) is a common serious health problem worldwide, which lacks efficient medical treatment. We aimed to develop and validate the machine learning (ML) models which could be used to the accurate screening of large number of people. This paper included 304,145 adults who have joined in the national physical examination and used their questionnaire and physical measurement parameters as model's candidate covariates. Absolute shrinkage and selection operator (LASSO) was used to feature selection from candidate covariates, then four ML algorithms were used to build the screening model for NAFLD, used a classifier with the best performance to output the importance score of the covariate in NAFLD. Among the four ML algorithms, XGBoost owned the best performance (accuracy = 0.880, precision = 0.801, recall = 0.894, F-1 = 0.882, and AUC = 0.951), and the importance ranking of covariates is accordingly BMI, age, waist circumference, gender, type 2 diabetes, gallbladder disease, smoking, hypertension, dietary status, physical activity, oil-loving and salt-loving. ML classifiers could help medical agencies achieve the early identification and classification of NAFLD, which is particularly useful for areas with poor economy, and the covariates' importance degree will be helpful to the prevention and treatment of NAFLD.

## Introduction

Non-alcoholic fatty liver disease (NAFLD) has become a sever public health problem worldwide (1, 2). The prevalence rate of NAFLD is around 20~30% and is increasing constantly. In the past 10 years, the prevalence rate of NAFLD has doubled (3). NAFLD is closely related to overweight or obesity, hyperlipidemia, type 2 diabetes mellitus (T2DM) and other chronic metabolic diseases: the prevalence of NAFLD is 60–90%, 27–92%, and 28–70% in obesity, hyperlipidemia and T2DM, respectively (4). NAFLD is a group of disease spectrum, the development of which is liver steatohepatitis, non-alcoholic steatohepatitis (NASH), liver fibrosis, cirrhosis, and even liver cancer. NAFLD is main cause leading to the fastest growing of liver cancer, and NASH has become the leading cause of liver failure in the United States (5–8). In recent years, the prevalence of NAFLD in China has gradually increased, and the prevalence has become younger: in 2014, a large sample meta-analysis reported that the prevalence of NAFLD in adults in mainland China was 20.09% (9). Therefore, the large-scale cohort or epidemiological study of NAFLD is of great significance. The implementation of the national physical examination encourages large-scale research, but a simple and easy method is still needed to classify NAFLD patients in the population.

Histologic biopsy is the gold standard for diagnosis of NAFLD, but it is invasive and requires high technology. Ultrasound, CT and MRI are the common diagnostic methods, but the cost of imaging examination is high when large-scale population screening. In order to facilitate the diagnosis of NAFLD, several predictive models have been introduced. Fatty liver index is an algorithm based on serum triglyceride and gamma glutamyl transferase (GGT) levels, body mass index (BMI) and waist circumference, which can predict liver steatosis in general population (10, 11). NAFLD liver fat score uses a formula including metabolic syndrome, T2DM, fasting serum insulin, aspartate aminotransferase (AST) and alanine aminotransferase (ALT) levels to estimate the percentage of liver fat content (12). SteatoTest is a logistic regression model of 12 predicting parameters: a2-macroglo-bulin (A2M), apolipoprotein A1 (ApoA1), haptoglobin, total bilirubin, GGT levels, cholesterol, triglycerides, glucose, age, gender and BMI (13). A prediction model based on laboratory includes six parameters: alanine aminotransferase, high-density lipoprotein cholesterol, triglyceride, hemoglobin A1c (HbA1c), white blood cell count and the presence of hypertension, and this model is used for the screening of NAFLD in common population (14). However, there is a problem that these prediction parameters are difficult to obtain. Although the existing NAFLD prediction models have been widely used, their application in large-scale epidemiological research and many areas of developing countries like China is limited.

It has been applied in medicine to establish accurate prediction model through machine learning. Machine learning outperforms conventional statistical methods with its ability to better identify variables relevant to clinical outcomes, better predictive performance, better modeling of complex relationships, ability to learn from multiple modules of data, and robustness to data noise. These tools have been used to diagnose fatty liver, meningitis, glaucoma, coronary heart disease, cancer and other diseases (14–21). Our purpose is to use machine learning to analyze the data of 304,145 physical examinees, and to establish a simple NAFLD screening model that does not rely on indicators tested in laboratory.

## Materials and Methods

### Study Population

The Chinese government provides free medical examinations for the people of Xinjiang. This data comes from the medical examination of Urumqi in 2018, consisting 643,439 cases. People who signed a written informed consent were eligible to participate in the study. Potential participants were excluded if they: (1) self-reported drinkers; (2) patients with specific diseases which can lead to fatty liver (3) age < 20; With a strict data filtration, 304,145 subjects contained in further analysis.

### Definition of NAFLD

The diagnosis of NAFLD was determined by the professionals of various physical examination institutions according to the standard of China Association of liver diseases (22). Patients are diagnosed with NAFLD when meeting the following three criteria: subjects without drinking or drinking history; no specific diseases leading to fatty liver such as viral hepatitis, liver disease induced by drug, total parenteral nutrition, hepatolenticular degeneration, and autoimmune liver disease; and Liver imaging of subjects was consistent with the diagnostic criteria for diffuse fatty liver. After summarizing all the results of physical examination, two doctors from the hepatology, department of a third-class hospital in Urumqi checked the diagnosis results of fatty liver, which were consistent with the preliminary diagnosis results.

### Variable Characteristics

There are three parts in NPE variables: questionnaire, physical examination and laboratory testing. The questionnaire has information about medical history, socioeconomics, and lifestyle (smoking, drinking, diet and exercise habits). Physical measurement indexes include height, body weight, heart rate and waist circumference. Laboratory test indicators include blood glucose, blood biochemistry and B-ultrasonic examination. In this study, we wanted to establish a simple model that can predict the risk of NAFLD without laboratory test variables. There were many missing values in NPE. We selected 17 variables with good data quality from the questionnaire and physical measurement parameters as candidate covariates (Table 1).

**Table 1:** Characteristics of variables.

**Characteristic**	**NAFLD** **(*N* = 58,654)**	**Normal** **(*N* = 245,490)**	***p*-value**
**Age (years)**	62 (50–71)	50 (40–65)	<0.001
**BMI (kg/m** ^**2**^ **)**	27.27(25.15–29.64)	23.71(21.91–25.80)	<0.001
**Waist circumference (cm)**	92(85.55–99)	84(78–90)	<0.001
**Ethnicity**, ***n*** **(%)**			<0.001
Han	38,132(65.01)	160,708(65.46)	
Uygur	8,973(15.30)	42,775(17.42)	
Kazak	1,317(2.25)	7,898(3.22)	
Hui	9,151(15.60)	27,843(11.34)	
Mongolian	98(0.17)	481(0.20)	
other nationalities	983(1.68)	5,785(2.36)	
**Gender**, ***n*** **(%)**			<0.001
Female	23,069(39.33)	104,083(42.40)	
Male	35,585(60.67)	141,407(57.60)	
**Physical activity,** ***n* (%)**			<0.001
Inactive	43,876(74.80)	149,349(60.84)	
Active	14,778(25.20)	96,141(39.16)	
**Career**			<0.001
Trader or service people	35,124(59.88)	180,260(73.43)	
Agriculture workers	19,268(32.85)	48,766(19.86)	
Factory workers	1,839(3.14)	6,230(2.54)	
Soldier	597(1.02)	1,058(0.43)	
Others	1,826(3.11)	9,176(3.74)	
**Smoking**			<0.001
No smoking	50,571(86.22)	225,638(91.91)	
0–20 cigarettes per day	6,119(10.43)	16,981(6.92)	
>20 cigarettes per day	1,964(3.35)	2,871(1.17)	
**Dietary status**, ***n*** **(%)**			<0.001
Meat based	55,034(93.83)	233,163(94.98)	
Meat balanced	1,980(3.38)	7,255(2.96)	
Vegetarian based	1,640(2.80)	5,072(2.07)	
**Sugar loving**, ***n*** **(%)**			<0.001
No	53,524(91.25)	233,709(95.20)	
Yes	5,130(8.75)	11,781(4.80)	
**Oil loving**, ***n*** **(%)**			<0.001
No	50,144(85.49)	232,123(94.55)	
Yes	8,510(14.51)	13,367(5.45)	
**Salt loving**, ***n*** **(%)**			<0.001
No	53,363(90.98)	235,452(95.91)	
Yes	5,291(9.02)	10,038(4.09)	
**Mental disease**, ***n*** **(%)**			<0.001
No	57,187(97.50)	240,826(98.10)	
Yes	1,467(2.50)	4,664(1.90)	
**Eye diseases**, ***n*** **(%)**			<0.001
No	55,545(94.70)	234,197(95.40)	
Yes	3,109(5.30)	11,293(4.60)	
**Gallbladder disease**, ***n*** **(%)**			<0.001
No	47,176(80.43)	227,057(92.49)	
Yes	11,478(19.57)	18,433(7.51)	
**T2DM**, ***n*** **(%)**			<0.001
No	38,136(65.02)	223,951(91.23)	
Yes	20,518(34.98)	21,539(8.77)	
**Hypertension**, ***n*** **(%)**			<0.001
No	37,127(63.30)	189,935(89.81)	
Yes	21,527(36.70)	55,555(10.19)	

*BMI, Body Mass Index; T2DM, type 2 diabetes mellitus*.

### Variable Definitions

Potential risk factors to evaluate NALFD contained: age, Body Mass Index (BMI), waist circumference, ethnicity, gender, physical activity, career, smoking, eating habits and some comorbidities.

Sociodemographic information, such as age (years), gender included “male” and “female”; ethnic groups were divided into six categories: “Han”, “Uygur”, “Kazak”, “Hui”, “Mongolian” and “other nationalities”; career included “Trader or service people”, “agriculture workers”, “factory workers”, “soldier” and “others”; the baseline comorbidities were mental diseases, eye diseases, gallbladder disease, T2DM, and hypertension (yes and no). The presence of eye diseases was defined as following: retinal hemorrhage, papilledema and cataract.

Lifestyle information includes smoking, physical activity and eating habits. Physical activity was defined as physical activity of at least 20 min per day (yes or no) in leisure time during the past 6 months (23); Individuals were defined as smokers if they had smoked at least one cigarette a day for at least 6 months (24). We also included daily smoking amount (0, 0–20 cigarettes, and >20 cigarettes). Dietary status included 3 options: “meat based”, “meat balanced”, “vegetarian based”, participants can choose one or more of them. Dietary hobby refers to whether participants are addicted to sugar, oil, or salt.

### Statistical Analysis

Data cleaning was performed first, and a descriptive analysis of the basic characteristics of the cleaned data was carried out. Categorical variables were expressed as numbers (percentages). Continuous variables conforming to normal distribution were expressed as mean ± standard deviation; Otherwise, the median and quartile were adopted. Chi-square or Fisher's exact test was used as appropriate to compare differences in categorical variables. The difference of *P* < 0.05 on both sides was considered statistically significant. Second, least absolute shrinkage and selection operator (LASSO) was used to filter variables, and the filtered variables were for subsequent model building. Third, because more normal subjects were included in this study than NAFLD subjects (an imbalanced class problem), the synthetic minority over-sampling technique (SMOTE) algorithm was used to solve this problem. Fourth, four machine learning models were constructed using the class-balanced data, and the performance of the models was compared. Finally, the variable importance ranking was carried out on the algorithm with the best model performance.

**Figure 1:**
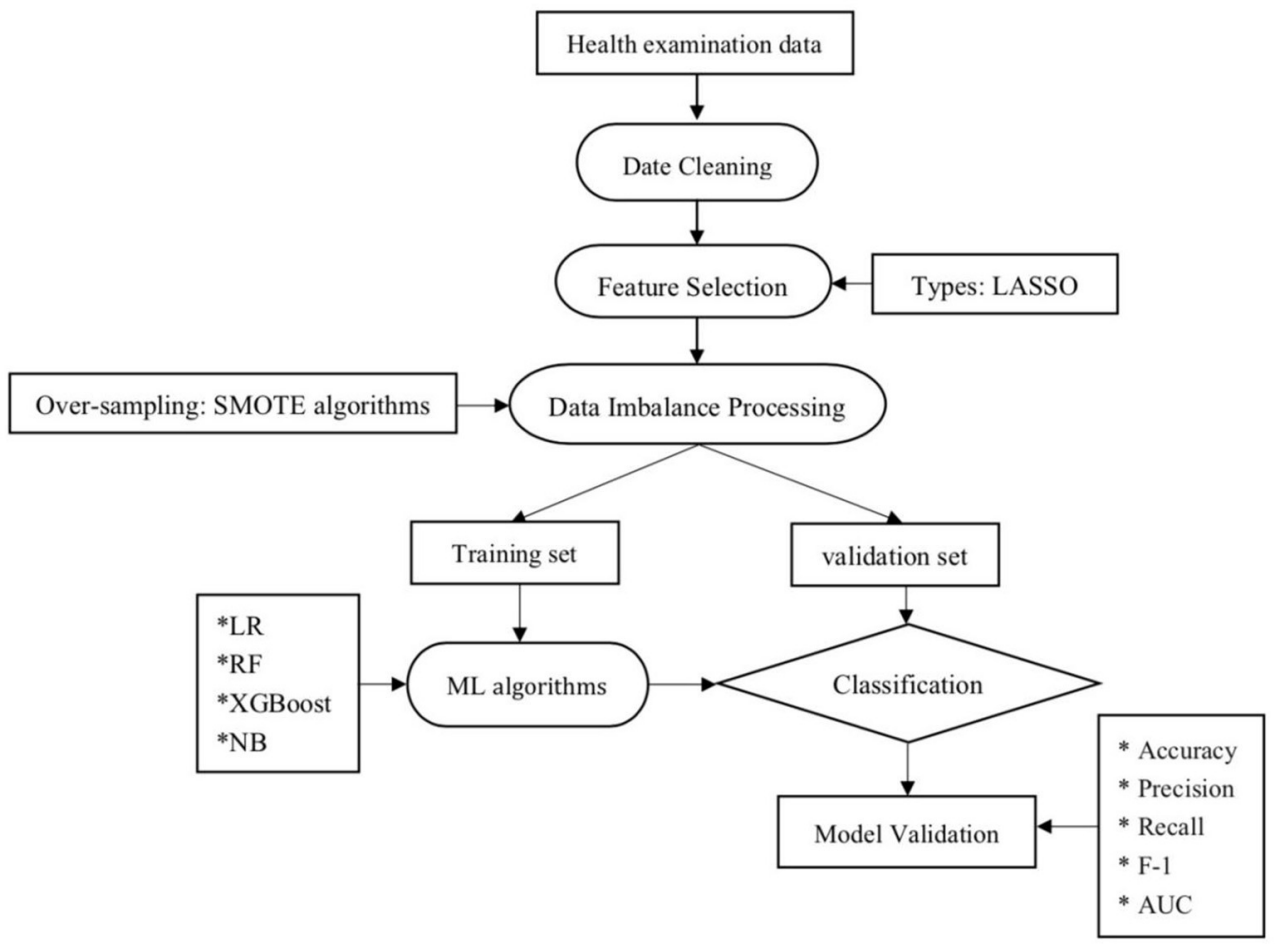
Machine learning flowchart of this study. LR, logistic regression; RF, random forest; NB, Naive Bayesian; ML, machine learning; LASSO, least absolute shrinkage and selection operator.

All data of cases including demographic and disease in the two groups were given in Table 1. The main objective of the ML techniques is to classify the NAFLD. The overview of the proposed ML algorithms has been shown in Figure 1.

#### Date Cleaning

NPE has a large amount of data, and the variables are chaotic, with a large number of missing values and outliers. Therefore, data preprocessing is a essential step (25). Firstly, we deleted nearly 200 variables that were not meaningful to this study. Secondly, we have made pre-processing of the nulls and outliers, deleting the variables with more than 20% nulls and imputing the variables otherwise. Besides, categorical variables were filled with the mode, and continuous variables were filled with the mean.

#### Feature Selection

For applying the LASSO penalized logistic regression as the approach to screen the risk factors. The purpose of this method was to minimize the LASSO cost function and to obtain all features with non-zero coefficients. The minimized objective function is:


$$\begin{array}{l} \underset{w}{\text{min}}\frac{1}{2n}\|Xw-y{\|}_{2}^{2}+\alpha \|w{\|}_{1} \end{array}$$

where *X* is a matrix of subject features, *y* is a vector of sample labels, *n* is the number of samples, *w* is a coefficient vector of the regression model, and α||*w*||_1_ is the LASSO penalty with the constant α and the ℓ_1_-norm of the coefficient vector ||*w*||_1_ (26).

#### Data Imbalance Processing

Normal subjects were more than subjects with NAFLD (an unbalanced-class problem). Generally, classes with few subjects are more difficult to predict than those with numerous subjects (27–30). The SMOTE algorithm was used to solve the negative impact of class imbalance, which belonged to the method of over-sampling, the principle of the method is to increase the number of a few classes of samples in classification to achieve sample balance, it is widely used as which can preserve important information in samples.

#### Classifier Comparison

Classification models were based on four popular supervised ML methods. For the linear model, the logistic regression model (LR) (31). For the decision tree approach, random forest (RF) model in the bagging method was used to combine multiple trees and the XGBoost model in boosting procedure was used to combine stumps of trees (32). Finally, Naive Bayesian (NB) Model which was based on probability (33).

#### Model Evaluation

The data set balanced by the SMOTE algorithm was randomly divided into training set 70% and validation set 30% (34, 35). The algorithms were compared based on confusion matrix and some indicators including accuracy, precision, recall, F-1 and receiver operating characteristic (ROC) (36). Several important measures, such as accuracy, precision, recall, F-1 could be calculated by using the confusion matrix.


$$\begin{array}{lll} Accuracy & = & \frac{TP+TN}{TP+TN+FP+FN}\;\\ Precision & = & \frac{TP}{TP+FN}\;\\ Recall & = & \frac{TP}{TP+FP}\;\\ F-1 & = & \frac{2\times Precision\times Recall}{Precision+Recall} \end{array}$$

#### Feature Importance Ranking

Tree-based models provide measures for variable importance. However, ML algorithms can not estimate an easy explanation number because the relationships that ML algorithms fitted are complex compared with regression models. Usually, this relationship is not directly summarized as any parameter, and there is no causal relationship or even statistical explanation (37). Instead, this measure can generally be thought of as ranking which variables are most “important” to the fitting model (38). Although variable importance ranking is not a substitute for target hypothesis testing for a given parameter, it can be used as a means of hypothesis generation to help identify factors worthy of further study and thus gain some insight into the factors influencing the prediction (39).

The software used in this study was python software version 3.7.2. “Pandas” library, “NumPy” library and “Matplotlib” library were used for null and outlier determination and interpolation, “Imlearn” library was used to solve data imbalance, and “Sklearn” library was used to establish ML models and verify the validation. LASSO penalized logistic regression by R statistical software version 3.3.2 “Glmnet” package.

## Results

### Patients and Variables

A total of 58,654 (19.3%) from the pool of 304,135 subjects was NAFLD. Each subject was composed of 17 kinds of variables (Table 1), it is observed that all attributes are highly statistically (*p* < 0.001) associated with NAFLD.

### Feature Selection

**Figure 2:**
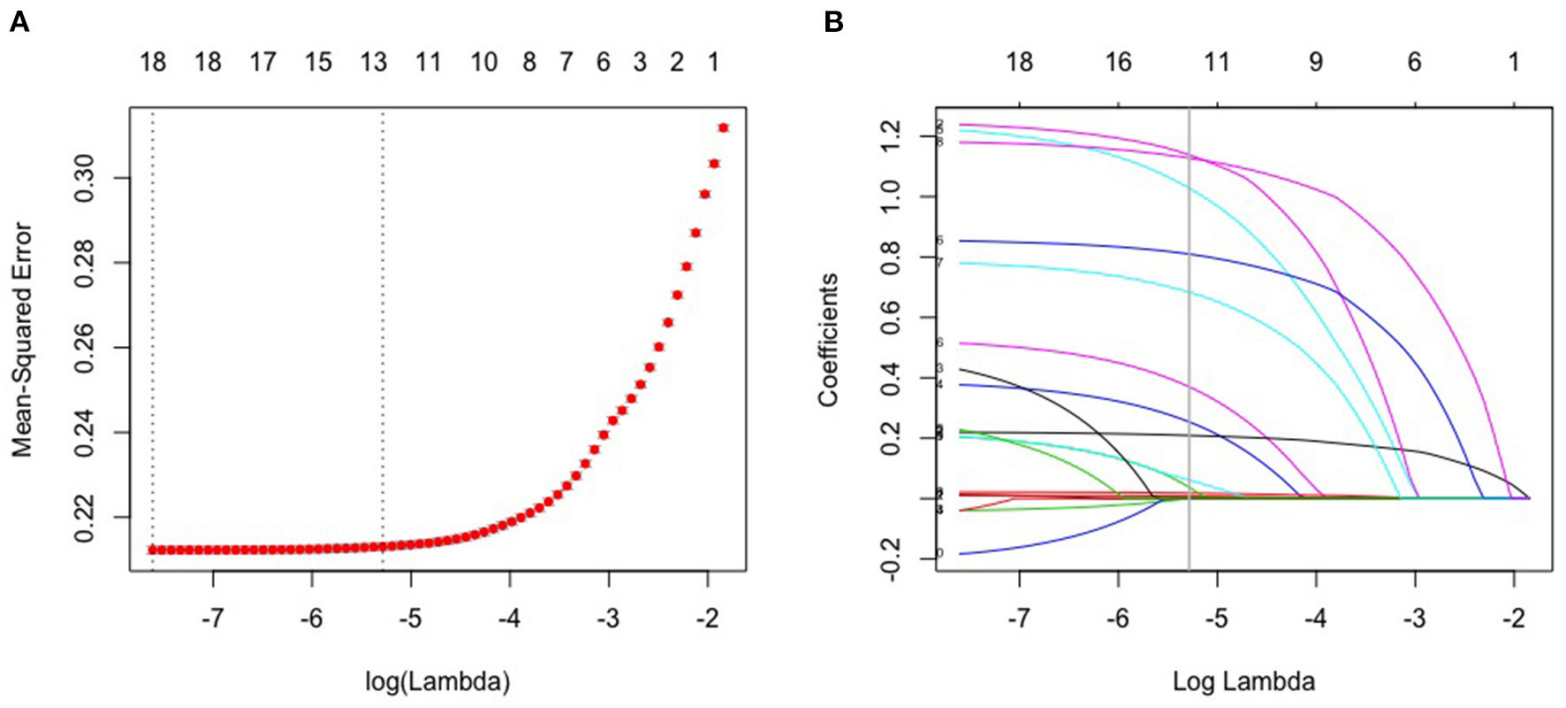
Lasso algorithm for feature selection. **(A)** mean-squared error (10-fold cross-validation criterion) of LASSO penalized logistic regression algorithm. **(B)** Vertical line was drawn at the value selected using 10 times cross-validation, where optimal lambda resulted in 12 features with nonzero coefficients.

Through LASSO regression, we got 12 non-zero coefficient characteristics, which showed that we reduced 17 indexes to 12 indexes. As it was shown in Figure 2. These features included age, gender, physical activity, smoking, BMI, waist circumference, dietary status, oil loving, salt loving, T2DM, gallbladder disease and hypertension. And these 12 indexes were for the subsequent construction of the model.

### Validation of the Validation Set

**Figure 3:**
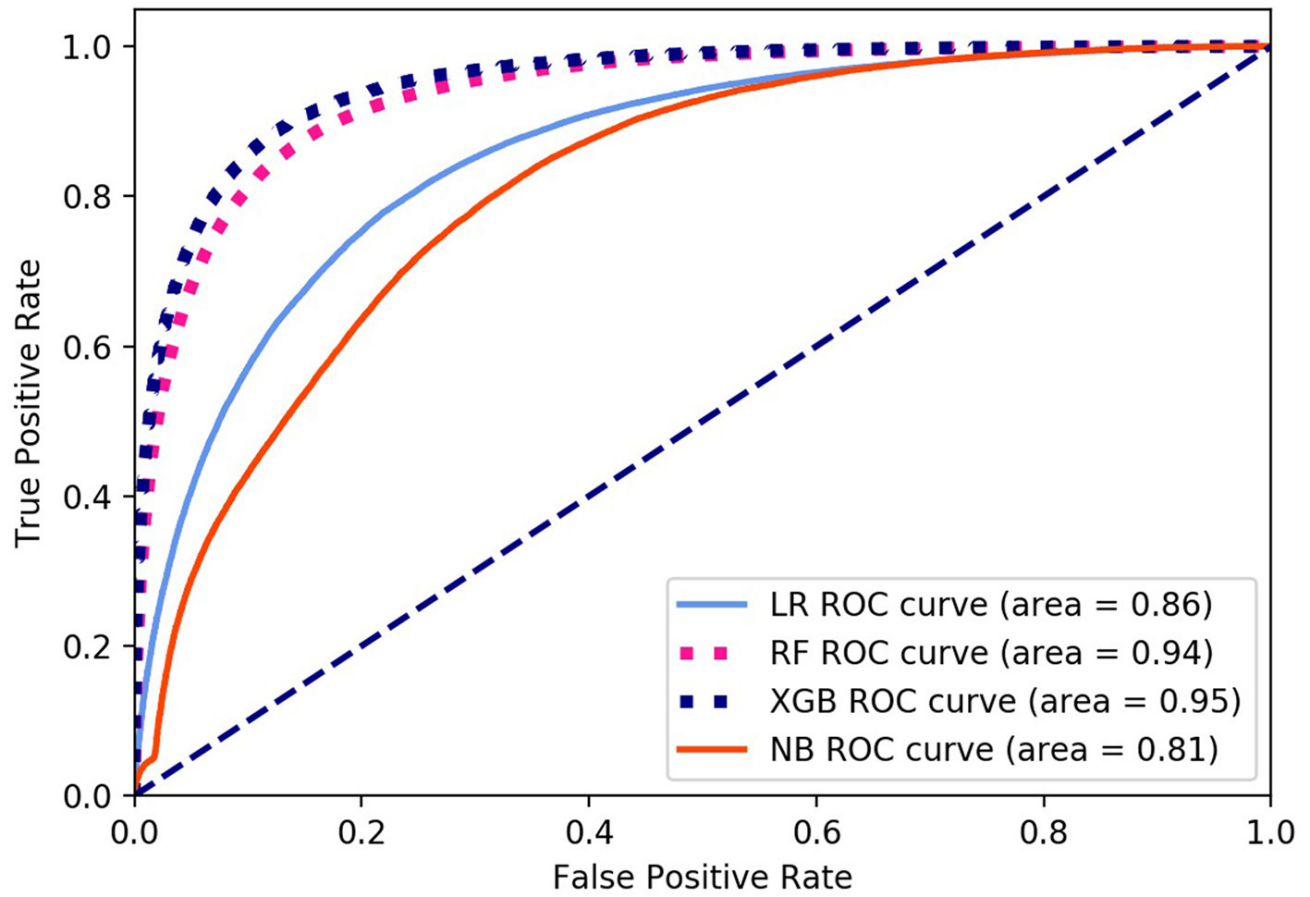
ROC curve of all algorithms. LR, logistic regression; RF, random forest; NB, Naive Bayesian; XGB, XGBoost.

Finally, we got 490,980 data sets consisting of 12 variables by SMOTE algorithm (Table 2), 343,686 subjects as the training set, and 147,294 subjects as the validation set. Our study has built four ML algorithms. Table 3 showed the performance of all classifiers. The confusion matrix has been displayed by Heatmap, the larger the number, the darker the color of the region, that is, the closer the color of TN and TP regions is to orange. On the contrary, the lighter the color of FN and FP regions are, the higher the accuracy of the classification model is. We got that the result of XGBoost was better than of the others (accuracy = 0.880, precision = 0.801, recall = 0.894, F-1 = 0.882, and AUC = 0.951). Figure 3 presented the ROC of all classifiers.

**Table 2:** Dataset description.

**Dataset**	**Samples distribution**	**Ratio**	**Description**
Original data	245,490/ 58,654	4:1	Original data with full instances
SMOTE data	245,490/ 245,490	1:1	Dataset is balanced utilizing SMOTE oversampling

**Table 3:** The results of classification algorithms.

**Model**	**Confusion matrix**	**Accuracy**	**Precision**	**Recall**	**F-1**	**AUC**
**LR**	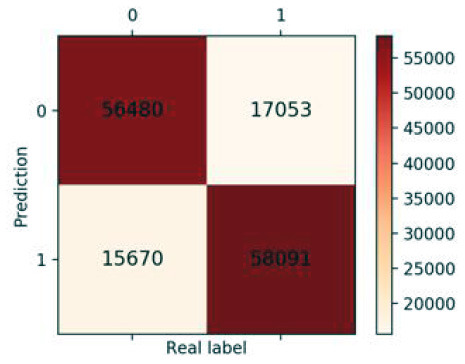	0.778	0.783	0.768	0.775	0.857
**RF**	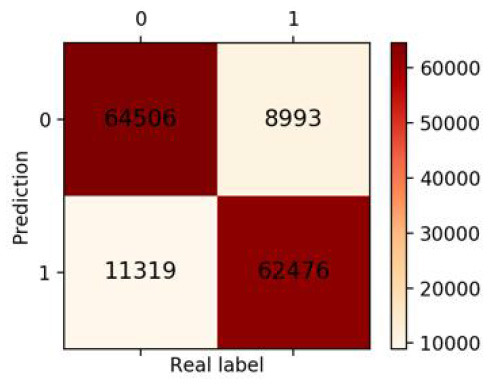	0.862	0.851	0.878	0.864	0.937
**XGBoost**	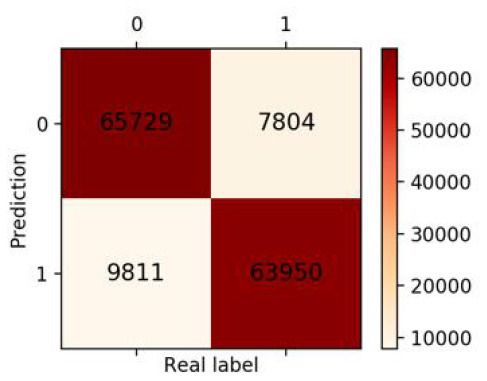	0.880	0.801	0.894	0.882	0.951
**NB**	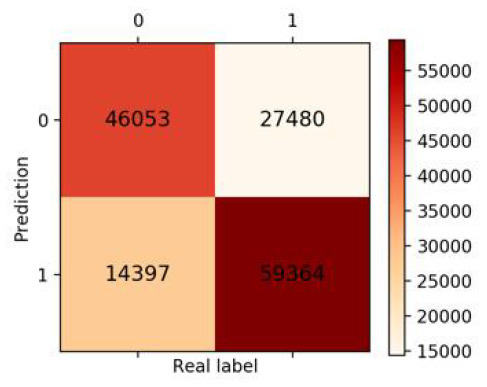	0.716	0.762	0.626	0.687	0.814

*AUC the area under the receiver operating characteristic (ROC) curve*.

*LR, logistics regression; RF, random forest; NB, naïve bayesian*.

### Variables Importance Ranking by XGBoost

**Figure 4:**
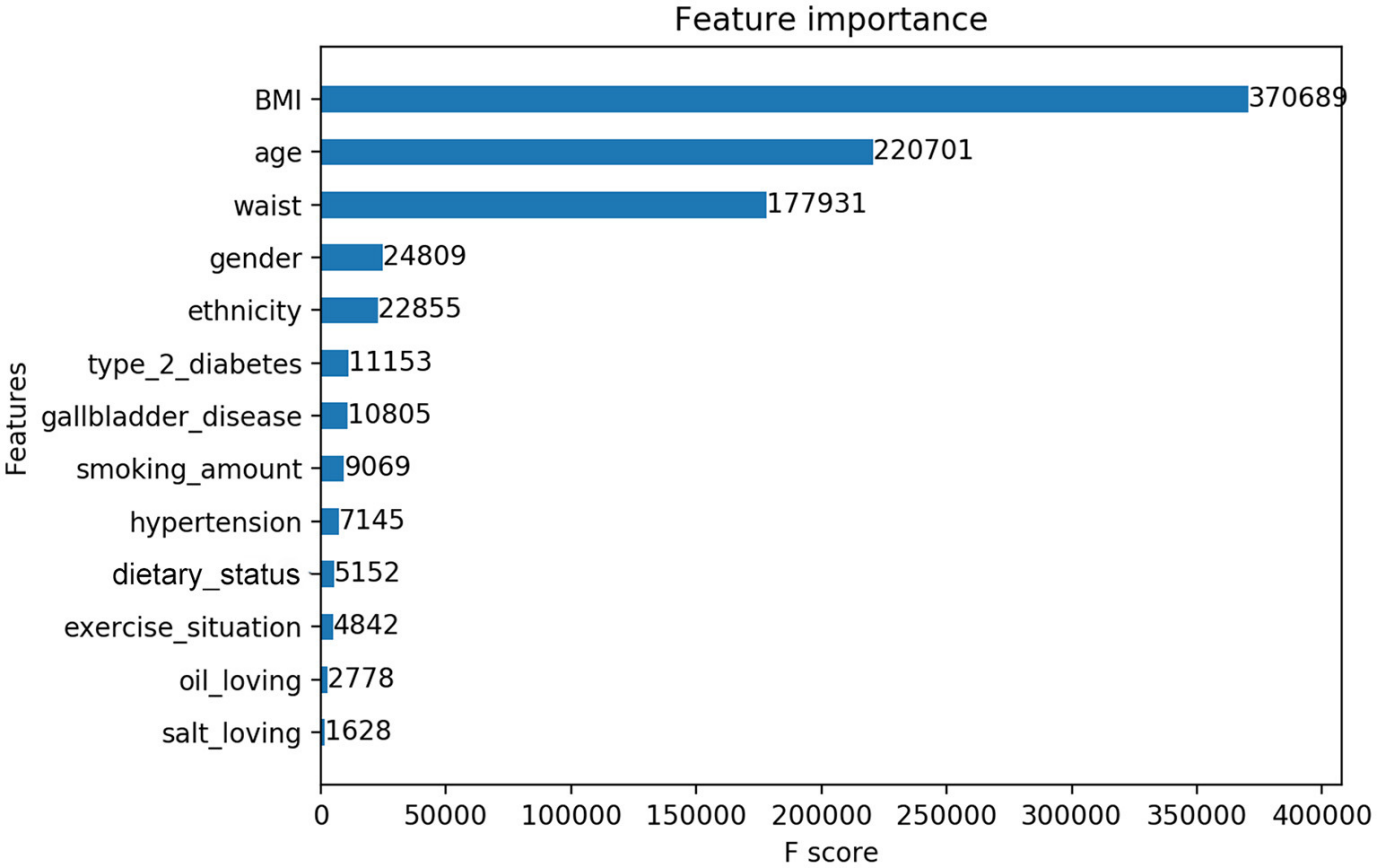
Feature importance contributed to the XGBoost model measured by F-score.

In this study, we output the results in terms of XGBoost model, who owned the best classification performance. XGBoost provided the importance score of each variable, attributing the predictive risk in 3 ways. Specifically, we chose the default method, which represents the relative number of times a variable is used to distribute data across all trees. There was only a small difference in the importance scores of the three methods, which did not affect the level of variable influence. The important measurement scores of the 12 variables are shown in Figure 4.

## Discussion

Non-alcoholic fatty liver disease (NAFLD) is the most common liver disease in the world, which is the main cause of liver cirrhosis and liver cancer. NAFLD lacks effective drug treatment, so early identification of disease and early prevention have the most effective means to improve the disease. In this study, through 12 questionnaires and physical measurement variables, four ML screening models based on 304,145 subjects for NAFLD in large-scale physical examination population were established, XGBoost got the best performance in the validation, which had accuracy = 0.880, precision = 0.801, recall = 0.894, F-1 = 0.882 and AUC = 0.951.

Detailed analysis of existing epidemiological data shows that the risk factors of NAFLD in China are similar to those in the West and other parts of Asia, metabolic syndrome (MetS) is associated with higher risk of non-alcoholic steatohepatitis and more progressive disease 0.2, In this study, BMI, waist, hypertension, gallbladder disease and T2DM were all the risk factors of MetS (40–42). On one hand, MetS is a strong predictor of NAFLD, while on the other hand, NAFLD is a good predictor for the clustering of components of MetS (43). In addition, a number of other risk factors for NAFLD have been identified in Chinese studies. These risk factors include advancing age, male gender, physical inactivity, high-fat intake, high-sugar intake, overeating, smoking, expanding waist circumference, and high-raising BMI (41, 42, 44). The conclusions of these studies were consistent with those of this study.

Our research has several advantages. First of all, some of the existing NAFLD prediction models involve laboratory and clinical parameters, and obtaining these parameters requires high human and financial resources, which limits the application of these models in large-scale epidemiological research and areas with poor health care level (10, 14, 45). All the variables in this study come from non-invasive and easily available measurement indicators and questionnaire indicators. This model can be applied to the prediction of NAFLD in the early stage and non-invasive, without expensive laboratory tests, especially in the areas with high epidemiological risk and low socio-economic status.

Secondly, this study is based on a large number of Chinese populations, which has a wide range of choices and is more extrapolated and representative. In addition, our data set covers many major ethnic groups in China, thus better assessing the characteristics of China's population.

Third, the occurrence and development of NAFLD are closely related to lifestyle, so improving lifestyle is an effective treatment (46). Our model not only can be used as a screening model for NAFLD, but also includes adjustable indicators such as diet, smoking, exercise, etc., which can guide people to prevent and delay the occurrence of disease through a healthy lifestyle. Although it is not clear whether exercise has independent benefits for NAFLD, exercise do can improve cardiovascular health, reduce weight, reduce peripheral, fat and liver insulin resistance.

Fourth, the analysis of NAFLD data is a challenging issue because most of the medical data are nonlinear, non-normal, correlation structured, and complex in nature. This study used LASSO penalized logistic regression vs. ML algorithms. LASSO works by shrinking the estimates of the regression coefficients and prevent overfitting due to collinearity of the covariates, which combines the advantages of selection process (easy to explain) and expression (robust), which is particularly useful in large data sets requiring efficient and fast algorithms (47–49). ML algorithms' outstanding performance in the field of processing complex data structures and big data makes it dominant in the field of healthcare and medical imaging, and compared with other machine learning methods, the performance of XGBoost can be improved more than 10 times (25, 50–53).

Surprisingly, compared with patients having non-NAFLD in previous studies, patients tend to eat a high calorie diet, especially in the form of carbohydrates and fats. Zelber Sagi et al. showed that NAFLD patients consumed more soft drinks and meat than the control group (54). Soft drinks contain a lot of sugar, and the intake of sugar is related to NAFLD (55). Musso et al. found that NAFLD patients had higher levels of saturated fat and cholesterol and lower levels of unsaturated fatty acids in their diet than healthy people (56). Although the ideal diet for NAFLD patients has not been determined, the data indicate that diet is important (57). However, in our study, we only got the weak effect of meat and vegetable combination, salt and oil preference on NAFLD (Figure 3), but not the effect of sugar preference on NAFLD. A possible reason for the irrelevance may be that the NPE diet survey was a cross-sectional study, with no professional evaluating the diet of the examined population. The main reason for the errors was that the self-reported eating habits of people undergoing physical examination were highly subjective and lack of professional evaluation indicators. Therefore, more accurate results can be obtained through follow-up of people's lives in future studies. Several limitations existed: firstly, previous studies confirmed that education and family history were important determinants of NAFLD, but we failed to obtain the education and family history of participants. Secondly, we lacked of objective and unified evaluation standard for some indicators, such as dietary status, which may reduce the accuracy of the prediction model. Thirdly, the data used in this study was the physical examination data of China, which might limit the extrapolation of the results. However, this study, based on a large sample of government, is one of the few literature studies providing NAFLD comprehensive epidemiological data for model development. Finally, the parameters in the dataset are not enough to compare with the scores of existing NAFLD prediction models. However, the purpose of this study is to provide a convenient and easily accessible model for the diagnosis of NAFLD through questionnaires and physical measurement. The results show that our model has high diagnostic accuracy and prediction ability.

## Conclusion

This study used a simple NAFLD screening model based on a large sample of 304,145 Chinese. The model can obtain high accuracy without relying on laboratory measurement parameters, especially in areas with poor economic conditions and high epidemiology.

## Data Availability Statement

The raw data supporting the conclusions of this article will be made available by the authors, without undue reservation.

## Ethics Statement

This study was performed in accordance with the principles outlined in the Declaration of Helsinki and approved by the Xinjiang Uygur Autonomous Region CDC Ethical Committee and the Institutional Review Board. People who signed a written informed consent were eligible to participate in the study.

## Author Contributions

MX and HY conceived the study. YZ and YW collected the data. MX and WJ performed the statistical analyses and drafted the manuscript. HY critically reviewed and edited the manuscript. All authors contributed to data analysis, drafting and revising the article, gave final approval of the version to be published, have agreed on the journal to which the article has been submitted, and agree to be accountable for all aspects of the work.

## Funding

This work was supported by the Region Social Science Foundation of Xinjiang (Grant No. 2021D01C238).

## Conflict of Interest

The authors declare that the research was conducted in the absence of any commercial or financial relationships that could be construed as a potential conflict of interest.

## Publisher's Note

All claims expressed in this article are solely those of the authors and do not necessarily represent those of their affiliated organizations, or those of the publisher, the editors and the reviewers. Any product that may be evaluated in this article, or claim that may be made by its manufacturer, is not guaranteed or endorsed by the publisher.
